# Polymeric Nanoparticles as Oral and Intranasal Peptide Vaccine Delivery Systems: The Role of Shape and Conjugation

**DOI:** 10.3390/vaccines12020198

**Published:** 2024-02-15

**Authors:** Prashamsa Koirala, Ahmed O. Shalash, Sung-Po R. Chen, Mohammad O. Faruck, Jingwen Wang, Waleed M. Hussein, Zeinab G. Khalil, Robert J. Capon, Michael J. Monteiro, Istvan Toth, Mariusz Skwarczynski

**Affiliations:** 1 School of Chemistry and Molecular Biosciences, The University of Queensland, Brisbane, QLD 4072, Australia; p.koirala@uq.net.au (P.K.); a.shalash@uq.net.au (A.O.S.); m.faruck@uq.net.au (M.O.F.); jingwen.wang1@uq.edu.au (J.W.); w.hussein@uq.edu.au (W.M.H.); 2 Australian Institute of Bioengineering and Nanotechnology, The University of Queensland, Brisbane, QLD 4072, Australia; sungpo.chen@uq.edu.au (S.-P.R.C.); m.monteiro@uq.edu.au (M.J.M.); 3 Institute for Molecular Bioscience, The University of Queensland, St Lucia, QLD 4072, Australia; z.khalil@uq.edu.au (Z.G.K.); r.capon@imb.uq.edu.au (R.J.C.); 4 School of Pharmacy, The University of Queensland, Woolloongabba, QLD 4102, Australia

**Keywords:** intranasal delivery, oral delivery, nanoparticles, vaccines, peptides, mucosal immunology, Group A *Streptococcus*, conjugates, physical mixture

## Abstract

Mucosal vaccines are highly attractive due to high patient compliance and their suitability for mass immunizations. However, all currently licensed mucosal vaccines are composed of attenuated/inactive whole microbes, which are associated with a variety of safety concerns. In contrast, modern subunit vaccines use minimal pathogenic components (antigens) that are safe but typically poorly immunogenic when delivered via mucosal administration. In this study, we demonstrated the utility of various functional polymer-based nanostructures as vaccine carriers. A Group A *Streptococcus* (GAS)-derived peptide antigen (**PJ8**) was selected in light of the recent global spread of invasive GAS infection. The vaccine candidates were prepared by either conjugation or physical mixing of **PJ8** with rod-, sphere-, worm-, and tadpole-shaped polymeric nanoparticles. The roles of nanoparticle shape and antigen conjugation in vaccine immunogenicity were demonstrated through the comparison of three distinct immunization pathways (subcutaneous, intranasal, and oral). No additional adjuvant or carrier was required to induce bactericidal immune responses even upon oral vaccine administration.

## 1. Introduction

Mucosal vaccination has a number of advantages over parenteral (intramuscular or subcutaneous) vaccination, including increased patient compliance, a lower chance of cross-infection or injury from contaminated needles, and the opportunity for self-administration, negating the need and expense for administration by trained medical staff [1,2,3]. Despite its many benefits, mucosal vaccine delivery is challenging. Intranasal delivery is limited due to the requirement for a relatively small antigen volume, minimal residence time at the nasal mucosal site, and poor absorption. Oral vaccination is challenged by the stomach’s extremely acidic environment and the digestive enzymes that are present throughout the gastrointestinal tract—particularly in the intestine—which typically lead to antigen degradation [4]. Furthermore, dose dilution inside the gastrointestinal tract and the viscous and thick mucus coating of the gastrointestinal epithelial cells hinder antigen absorption, requiring drastic vaccine dose increases to achieve the desired immunogenicity. The need for multiple doses is not only more costly and inconvenient for administration but also increases the risk of the development of oral tolerance against a given antigen. As a result, there is significant demand for a mucosal delivery system that can protect antigens from degradation while also triggering strong immune responses.

Group A *Streptococcus* (GAS, *Streptococcus pyogenes*) is a Gram-positive bacterium that infects the skin and throat and can lead to invasive diseases, such as pneumonia, necrotizing fasciitis, and streptococcal toxic shock, in addition to deadly autoimmune diseases, including rheumatic fever and rheumatic heart disease (RHD), which is the major cause of cardiac death in children and young adults [5]. It affects over 33 million individuals worldwide with annual mortality exceeding 300,000 [6]. There has been a sharp rise in invasive GAS (iGAS) cases reported in Europe lately [7]. Compared to the pre-COVID era (2017–2019), the rate of pleural empyema in children in Scotland that required the insertion of a chest drain had risen by 95% by late 2022. Remarkably, this was correlated with iGAS infection rather than *Streptococcus pneumoniae* [8]. A surge in severe cases of GAS disease has also been observed in Asia, Australia, and the USA [9,10]. A vaccine is needed to effectively combat iGAS and prevent the development of life-threatening GAS post-infection complications [11].

Here, we propose the use of polymer-based antigen delivery systems to develop a mucosally active GAS vaccine. We recently proved that polymeric nanoparticles built from polystyrene-poly(*N*-isopropylacrylamide)-poly(*N*-dimethylacrylamide) can enhance the immunogenicity of peptide antigens following parenteral (subcutaneous) immunization [12]. However, the ability of these polymers to act as mucosal delivery systems was not examined. Therefore, a series of vaccine candidates was prepared by conjugating or mixing a GAS antigen (**PJ8**) with different shapes of polymeric nanoparticles (including **rods**, **worms**, **spheres**, and **tadpoles**). **Sphere–** and **tadpole–PJ8** conjugates were not developed previously and have not been reported. The **PJ8** was composed of GAS M protein-derived conserved B-cell epitope J8 (QAEDKVKQSREAKKQVEKALKQLEDKVQ) [13] conjugated to universal T-helper cell epitope PADRE (AKFVAAWTLKAAA) [14], which has been often used to improve the efficacy of humoral immune responses. Both these epitopes have been proven safe and effective in clinical trials [15,16]. All vaccine candidates were then analyzed for their ability to induce antibody production upon both intranasal and oral delivery. Finally, the bactericidal efficacy of the produced antibodies was examined.

## 2. Results

### 2.1. Synthesis and Physiochemical Characterization

**Figure 1:**
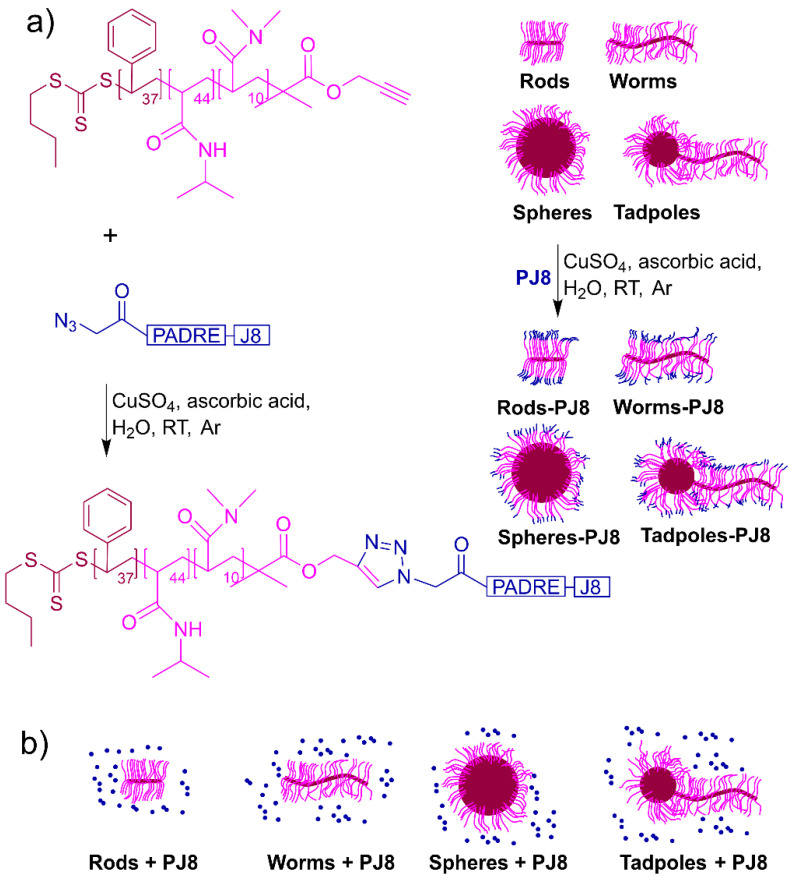
Polymeric nanoparticle-based vaccine candidates. Three components were used to produce vaccine candidates: B-cell epitope (J8), T-helper epitope (PADRE), and polymeric units. These were combined either by (**a**) conjugation reaction to produce **rods–PJ8**, **worms–PJ8**, **spheres–PJ8**, and **tadpoles–PJ8,** or (**b**) physical mixing of antigen and polymeric units to produce **rods + PJ8**, **worms + PJ8**, **spheres + PJ8**, and **tadpoles + PJ8**. The symbolization for conjugates is ‘–’, whereas that for physical mixers is ‘+’. The antigen PJ8 is shown in a blue font.

As previously reported, the peptide, **PJ8**, was synthesized manually using Fmoc-SPPS and derivatized with azide moieties on its N-terminus end [12]. By employing copper(I)-catalyzed alkyne–azide cycloaddition, azide-functionalized **PJ8** was conjugated to alkyne-substituted **spheres** and **tadpoles** to produce **spheres–PJ8** and **tadpoles–PJ8**, respectively (Figure 1). **Worms–PJ8** and **rods–PJ8** were produced according to reported protocol [12]. To confirm the completion of conjugation, the conjugates were assessed via UV-VIS spectroscopy, ninhydrin assay, and IR spectroscopy (Supporting Information, Figures S1–S3). The amounts of **PJ8** conjugated to rod-, worm-, sphere-, and tadpole-shaped polymeric nanoparticles were quantified using a BCA protein assay. A relatively high substitution rate was achieved (~72%) for all conjugates (Supporting Information, Figure S4). The size and morphology of physically mixed and conjugated nanoparticles were analyzed by DLS (Supporting Information, Table S1, Figure S5) and TEM (Supporting Information, Figure S6). Particle size and dispersion homogeneity observed through TEM correlated well with the size and PDI measured by DLS; namely **spheres** (alone, in mixture with antigen and conjugated to antigen) were approximately 500 nm in diameter; **rods** were 70 nm in diameter and 500 nm in length; **worms** were 70 nm in diameter and a few micrometers in length; and **tadpoles** were 200 nm in diameter (head) and 1 µm in length (tail). Neither physically mixing nor conjugating **PJ8** to **rods**, **worms**, **spheres**, or **tadpoles** altered the particles’ structure.

### 2.2. Evaluation of Antibody Responses upon Oral Vaccine Delivery

Conjugates, **rods–PJ8**, **worms–PJ8**, **spheres–PJ8**, and **tadpoles–PJ8**, were administered intranasally and orally to mice (n = 5). Physical mixtures, **rods + PJ8**, **worms + PJ8**, **spheres + PJ8**, and **tadpoles + PJ8**, were prepared using the same amount of **PJ8** and polymeric nanoparticles as the conjugates and were delivered into mice identically as the conjugates. Due to the ability to induce strong mucosal immune responses against a variety of antigens, including **PJ8**-based vaccine candidates, CTB was selected as a positive control (**PJ8**+CTB) [17,18]. All mice received 30 µg of **PJ8**, irrespective of the antigen’s conjugation or physical mixing.

**Figure 2:**
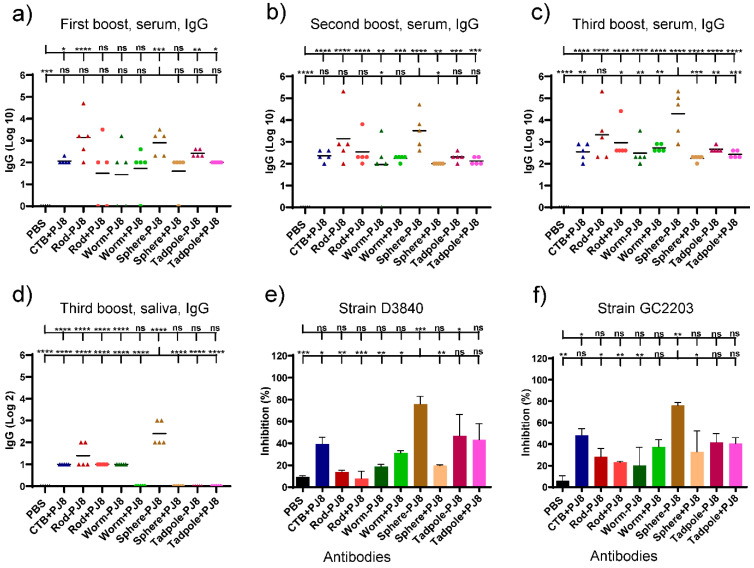
Immune responses following oral immunization of C57BL/6 mice (n = 5 per group) with PBS (negative control), **PJ8 + CTB** (adjuvanted control), and experimental conjugates and physical mixtures. J8-specific serum (**a**–**c**) and saliva (**d**) IgG titers in individual mice are shown by each point, and the average antigen-specific IgG titers are shown by the bars. (**e**,**f**) Average bactericidal activity of immunized mouse sera against different GAS strains (GC2203 and D3840). One-way ANOVA with Tukey’s multiple comparison test was used for statistical analysis. (*) *p* < 0.05, (**) *p* < 0.01, (***) *p* < 0.001, (****) *p* < 0.0001. The statistical analysis is presented in comparison to PBS (upper line) and **sphere–PJ8** (lower line).

Four vaccination doses were given to mice that were orally immunized at two weeks intervals. Enzyme-linked immunosorbent assays (ELISA) were used to measure the total J8-specific IgG titers in the blood and saliva (Figure 2a–d). The mixtures, **rods + PJ8**, **worms + PJ8**, **spheres + PJ8**, and **tadpoles + PJ8**, induced lower antibody response levels against J8 than their corresponding conjugates, even after the third boost. Serum IgG levels markedly increased when **PJ8** was conjugated with polymeric nanoparticulate carrier systems. The highest J8-specific IgG titers from the serum were detected upon immunization with **spheres–PJ8**, followed by **rods–PJ8**. Remarkably, **spheres–PJ8** induced significantly higher immune responses than the adjuvanted positive control, **PJ8**+CTB (*p* < 0.01). The conjugates, **worms–PJ8** and **tadpoles–PJ8**, along with all of the physical mixtures, showed similar levels of antibody titers. No statistical differences in antibody titers in the serum were observed compared to CTB-adjuvanted **PJ8** (Figure 2a–c). Salivary IgG levels of mice immunized with **spheres–PJ8** were relatively low but higher than those induced by the positive control (Figure 2d). Following oral vaccination, neither the control group nor any of the vaccine candidates were able to trigger the development of J8-specific immunoglobulin A (IgA) antibodies in saliva (data not shown).

Antibodies produced by mice following the last oral immunization were tested for their bactericidal ability against different strains of GAS clinical isolates (D3840, GC203) (Figure 2e,f). Among all the sera from mice treated with conjugates and mixtures, **spheres–PJ8** sera showed the most apparent bactericidal activity.

### 2.3. Evaluation of Antibody Responses upon Nasal Vaccine Delivery

**Figure 3:**
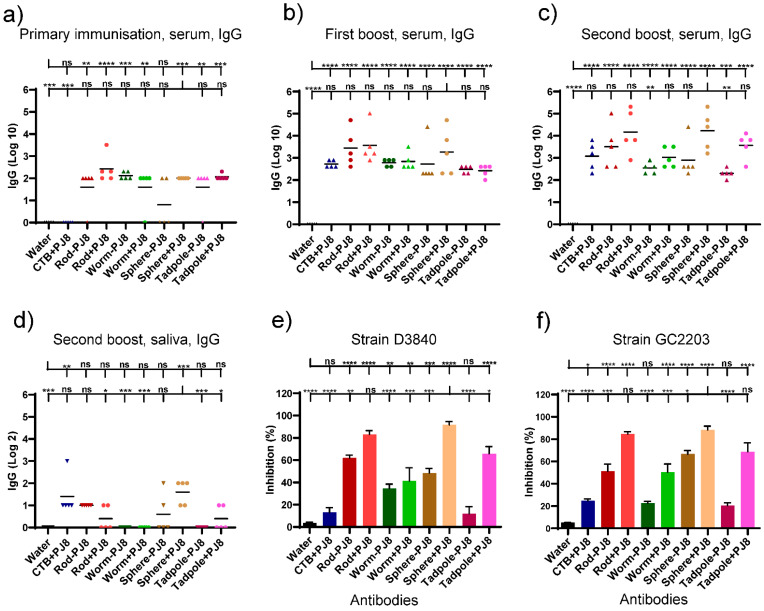
Immune responses in C57BL/6 mice (n = 5 per group) after intranasal injection of water (negative control), PJ8+CTB (adjuvanted control), and experimental conjugates and physical mixers. J8-specific serum (**a**–**c**) and saliva (**d**) IgG titers. An individual mouse is represented by each point, and average antigen-specific IgG titers are shown by the bars. (**e**,**f**) Average percentage of bactericidal activity against two different GAS strains (GC2 203 and D3840) based on serum collected 41 days after the initial intranasal vaccination. One-way ANOVA with Tukey’s multiple comparison test was used for statistical analysis. (*) *p* < 0.05, (**) *p* < 0.01, (***) *p* < 0.001, (****) *p* < 0.0001. The statistical analysis is presented in comparison to PBS (upper line) and **sphere + PJ8** (lower line).

A second cohort of mice (n = 5 per group) was used to assess the intranasal delivery of the conjugates and physical mixtures of the nanoparticles. Intranasally administered **PJ8** adjuvanted with CTB was used as a positive control. Mice were intranasally immunized three times at two weeks intervals. Post-immunization serum and saliva IgG titers were determined by ELISA (Figure 3a–d). The highest J8-specific IgG titers were detected in the blood of mice immunized with physical mixtures, **rods + PJ8** and **spheres + PJ8**. The conjugates, **rods–PJ8**, **worms–PJ8**, **spheres–PJ8**, and **tadpoles–PJ8**, induced lower antibody response levels against J8 than their corresponding mixtures after three immunizations; however, the differences were not statistically significant. Saliva was also examined for the generation of J8-specific IgG antibodies. Among all vaccine candidates, only **spheres + PJ8** was able to induce significant mucosal antibody production (*p* < 0.001, Figure 3d). Following intranasal immunization, neither the control group nor any of the vaccine candidates were able to produce J8-specific IgA in saliva (data not shown).

Antibodies produced by the immunized mice were tested for their ability to kill different strains of GAS clinical isolates (D3840, GC2203) (Figure 3e,f). Physical mixtures, **rods + PJ8** and **spheres + PJ8**, showed the highest levels of bactericidal activity against both GAS strains. All sera immunized with conjugates were less bactericidal than the mixtures, showing the same trend as IgG titers.

### 2.4. Correlates of Immunity

**Figure 4:**
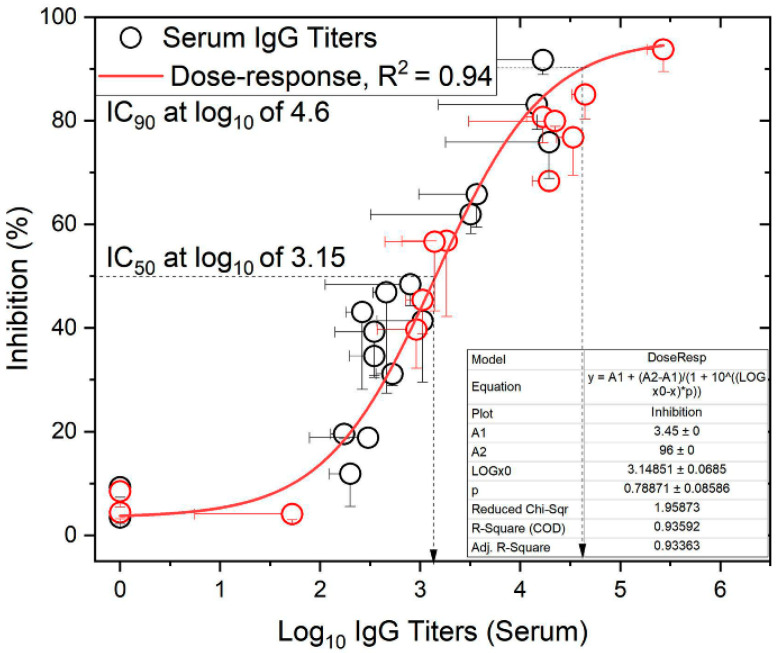
The relationship between anti-J8 IgG titers in mouse sera and bactericidal activity against cultured GAS bacteria (D3840 strain). The data were fitted to a sigmoidal relationship (R^2^ = 0.94). Red circles denote subcutaneous immunization [12], while black circles denote intranasal and oral immunization. Dashed arrows represent the interpolated IC_50_ and IC_90_ titer values from the fitted curve.

Previously reported serum J8-specific IgG titers following subcutaneous immunizations [12], and the data reported here from intranasal and oral immunizations, correlated with bactericidal activity against GAS clinical isolates. The relationship between bactericidal activity and serum anti-J8 IgG titers fitted a sigmoidal function, with R^2^ = 0.94 against the GAS strain D3840 (Figure 4) and R^2^ = 0.88 against GC2203 (Supporting Information, Figure S7). The fit against the GAS strain D3840 gave an IC_50_, i.e., log_10_ titers at 50% inhibition/bactericidal activity, of 3.15, whereas the IC_90_ was log_10_ of 4.6. Similarly, the fit against GAS GC2203 gave IC_50_ and IC_90_ values of 3.3 and 5.2, respectively. These demonstrate the required titers to inhibit bacterial growth at the employed bacterium inoculum CFU/mL.

### 2.5. Enzymatic Stability In Vitro

**Figure 5:**
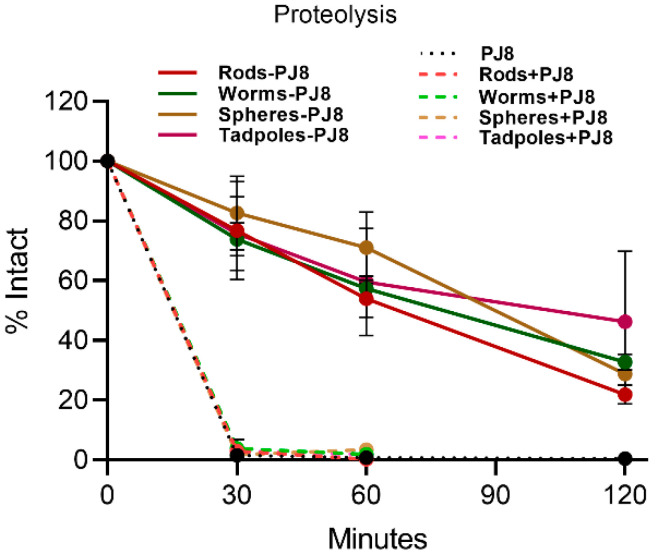
In vitro proteolysis stability of antigen (**PJ8**) alone, in physical mixtures, and in conjugates against bovine trypsin.

The proteolytic stability of physical mixtures and the conjugates was examined against treatment with bovine trypsin at 37 °C (Figure 5). The conjugates (**rods–PJ8**, **worms–PJ8**, **spheres–PJ8**, and **tadpoles–PJ8**) were fairly resistant to proteolysis (17–26% degradation after 30 min), while antigen **PJ8** alone and physically combined with nanoparticles (**rods + PJ8**, **worms + PJ8**, **spheres + PJ8**, and **tadpoles + PJ8**) fully degraded by trypsin within 30 min. The conjugates remained 22–46% intact after two hours.

## 3. Discussion

Subunit vaccines delivered as nanoparticles are presented to the immune system more effectively, shielded from enzymatic breakdown, and able to elicit immunological responses without the requirement for an adjuvant [19]. Thus, we recently developed novel self-adjuvant vaccine delivery systems based on polymeric nanoparticles of various shapes, including **rods**, **worms**, **spheres**, and **tadpoles** [12]. Delivered subcutaneously, these nanoparticles physically mixed or conjugated with a peptide epitope (**PJ8**) induced high antibody titers without the need for an adjuvant. Significantly, the mixture, **rods + PJ8**, triggered the production of even higher IgG titers than **PJ8** adjuvanted with very powerful, but toxic, complete Freund’s adjuvant. The other particle shapes were less effective in stimulating immune responses. Interestingly, nanoparticle shape also influenced the efficacy of orally delivered vaccines. When delivered orally in a *Necator americanus* hookworm challenge study in mice, rod-shaped nanoparticles (20 nm wide and 100–500 nm long) were more effective than spherical (300 nm) nanoparticles in an aspartic protease *Na*-APR-1-derived vaccine [20]. In addition, rod-shaped mesoporous silica nanoparticles (100 × 250 nm) were more effectively taken up via oral routes than spherical particles (250 nm) [21]. Yu et al. examined the mucosal penetration properties of mesoporous silica nanospheres and nanorods, demonstrating that rod-like mesoporous silica (80 × 240 nm) diffused intestinal mucus more efficiently than spheres (80 nm) [22]. Likewise, Banerjee et al. reported that rod-like nanoparticles (150 × 450 nm) were more effective than spheres (~200 nm) in terms of mucosal penetrating ability and cellular absorption, possibly as a result of a larger contact surface area to volume ratio allowing for significant interaction and adherence with cell membranes [23].

Thus, **rods + PJ8**, and possibly **rods–PJ8**, could potentially produce the most significant immune responses. However, in the current study, mice treated orally with the newly developed **spheres–PJ8** conjugate (~500 nm) produced higher levels of systemic IgG than **rods–PJ8** (~20 × 100–500 nm according to TEM), although they were equipotent after the first boost (Figure 2). As the **rods** were relatively thin, the **spheres** had a larger surface area, which may explain their higher potency. Importantly, serum from mice immunized with **spheres–PJ8** was bactericidal. All other polymeric nanoparticles conjugated or mixed with **PJ8**, as well as the positive control (CTB-adjuvanted **PJ8**), induced relatively low IgG titers. The weak immunogenicity of **PJ8**/CTB may be related to CTB’s ability to trigger tolerance upon oral administration [24,25]. Interestingly, physical mixtures of **rods**, **worms**, **spheres**, **tadpoles**, and **PJ8** generated considerably lower systemic IgG titers than the corresponding conjugates in mice upon oral immunization. The poor immune-stimulating capability of the physical mixtures was clearly related to antigen instability against enzymatic degradation (Figure 5). Notably, stability increased greatly when **PJ8** was conjugated to the nanoparticles. Indeed, we have shown that a variety of nanoparticles conjugated to peptide antigens can deliver effective immune stimulation in vivo upon oral vaccine administration [18,20,25,26].

Intranasal immunization is attractive as it mimics the natural route of GAS infection. Moreover, the physicochemical conditions of the nasal cavity are less severe than those of the gastrointestinal tract. Other advantages of intranasal vaccination include ease of self-administration, smaller doses compared to oral vaccination, and a highly vascular mucosal region for antigen uptake [27]. Yet, challenges to this strategy have hindered outcomes. Antigen removal through nasal mucociliary movement is still a concern, as are tight connections between epithelial cells, keratinized epithelium, and nasal enzymes [27,28,29]. Consequentially, suitable delivery systems and adjuvants must be utilized to optimize the efficacy of intranasal vaccination. Various nanoparticles have been administered intranasally with a natural polymer; chitosan-coated nanoliposome vaccines have proven particularly efficient [30]. For example, methylated chitosan-derivative nanoparticles were very effective in inducing humoral immune responses upon intranasal delivery; however, conjugation of the antigen to the nanoparticles was required for optimal efficacy [31,32]. Among synthetic polymers, polyacrylic nanoparticulate delivery systems and polyelectrolyte complexes were highly immunogenic, but only when the antigen was conjugated to, encapsulated, or entrapped within polymeric nanoparticles [33,34]. Here, we demonstrated that intranasal administration of physical mixtures of **rod**, **worm**, **sphere**, and **tadpole** polymeric nanoparticles and the **PJ8** antigen induced the production of stronger systemic IgG responses compared to the corresponding conjugates; however, the differences were not statistically significant (Figure 3). Remarkably, **spheres + PJ8** and **rods + PJ8** induced the highest and most bactericidal systemic IgG responses when administered intranasally. Thus, in contrast to oral delivery, antigen conjugation was not required for mucosal activity of polymeric vaccine delivery systems. Intranasal antigen delivery also required fewer immunizations compared to oral delivery to trigger similar IgG titers in mice. These differences can be explained by the fact that nasal mucosa has less proteolytic activity than oral mucosa [35], allowing for a smaller vaccine dose to produce the same immunological response and eliminating the need for antigen conjugation.

Importantly for further GAS vaccine development, the levels of induced antibody titers correlated well (R^2^ of 0.94) with the antibody’s ability to kill clinical GAS isolates, regardless of immunization route (subcutaneous, intranasal, or oral) (Figure 4). It is also important to note that these vaccine candidates can be freeze-dried and resuspended immediately prior to administration for ease of vaccine manufacture, storage, and distribution.

## 4. Conclusions

We demonstrated that nanoparticle shape and conjugation to an antigen have an important influence on the humoral immune responses generated. Spherical and rod-shaped nanoparticles were preferred for intranasal immunization over worms and tadpoles; spherical nanoparticles were most effective when administered orally, while rods proved best when administered subcutaneously. Antigen–nanoparticle conjugation was required for oral vaccine delivery but was not preferable for intranasal delivery, while the physical mixture was more effective for subcutaneously delivered vaccines. Overall, we have demonstrated that nanoparticle-based vaccines must be tailored to the chosen delivery route. Significantly, regardless of the delivery route, nanovaccines can be highly effective in stimulating antibody production capable of eliminating GAS bacteria. Our strategy of developing a self-adjuvanting polymeric nanoparticle-based delivery system for mucosal delivery of a GAS vaccine paves the way for oral and intranasal delivery of subunit vaccines against a variety of infectious diseases.

## 5. Experimental Section

### 5.1. Materials and Equipment

All solvents and chemicals utilized were commercially available and synthesis grade, unless otherwise specified. The following materials were purchased from Merck (Hohenbrunn, Germany): *N*,*N*-diisopropylethylamine, rink-amide 4-methylbenzhydrylamine resin (substitution value 0.52 mmole/g), ethanol, acetone, *N*,*N*-dimethylformamide, piperidine, acetic anhydride, diethyl ether, acetonitrile, triisopropylsilane, dichloromethane, ethyl acetate, methanol, triisopropylsilane, trifluoroacetic acid (TFA), and yeast extract. 1-[6-bis(dimethylamino)methylene]-1*H*-1,2,3-triazole[4,5-b]pyridinium-3-oxide hexafluorophosphate, copper sulfate pentahydrate, and fluorenylmethyloxycarbonyl (Fmoc)-protected L-amino acids were acquired from Merck Chemicals (Darmstadt, Germany), Novabiochem (Darmstadt, Germany), and Mimotopes (Melbourne, Australia). Alpha Aesar (Ward Hill, MA, USA) provided 4-pentynoic acid. Copper wires, 2-azidoacetic acid, and phosphate-buffered saline (PBS) were purchased from Sigma-Aldrich (St. Louis, MO, USA). The snakeskin pleated dialysis tube and Micro BCA protein testing kit were supplied by Thermo Scientific (Scoresby, VIC, Australia). L-ascorbic acid and sodium hydrogen carbonate were purchased from Ajax Finechem and Chem-Supply, respectively. Complete Freund’s adjuvant and goat anti-mouse IgG (H+L)–HRP (horseradish peroxidase) conjugate were procured from Millipore (Temecula, CA, USA); analytical-grade Tween 20 was acquired from VWR International (Tingalpa, QLD, Australia). We purchased goat anti-mouse IgG (H+L)-HRP conjugate secondary antibody from Bio-Rad (Gladesville, NSW, Australia). O-phenylenediamine dihydrochloride tablets and trypsin from the bovine pancreas were purchased from Sigma-Aldrich (Castle Hill, NSW, Australia). We purchased 96-well micro test plates (high binding, polystyrene, flat base) from Sarstedt (Mawson Lakes, SA, Australia). Merck (Kilsyth, VIC, Australia) provided the skim milk powder. Sigma-Aldrich (St. Louis, MO, USA) provided the pyridine and potassium cyanide, while Merck (Hohenbrunn, Germany) provided the phenol. Monteiro and coworkers synthesized rod-, worm-, spherical, and tadpole-shaped polymeric nanoparticles [36,37,38].

An APE Sciex API3000 triple quadrupole mass spectrometer (Ontario, Canada) and Shimadzu apparatus (Kyoto, Japan) were used for electrospray ionization mass spectrometry (ESI-MS) utilizing LabSolutions 5.51 software. A Shimadzu (Kyoto, Japan) instrument was used in conjunction with a C18 analytical Vydac column (4.6 mm × 250 mm, 218TP54; 5 m) and solvents B (0.1% TFA in 90:10 acetonitrile/water) and A (0.1% TFA in MilliQ water), at a flow rate of 1 mL/min and UV absorbance of 214 nm, to achieve analytical reverse-phase high-performance liquid chromatography (RP-HPLC). For purification, Shimadzu preparative RP-HPLC apparatus (LC-20AP × 2, CBM-20A, SPD-20A, and FRC-10A) was utilized. A C18 column (218TP1022; 10 m, 22 × 250 mm) with a UV absorbance of 214 nm was utilized, and a flow rate of 20.0 mL/min was employed. Dynamic light scattering (DLS) measurements were obtained using dispersion technology software from a Zetasizer Nano ZP instrument purchased from Malvern Instruments (Malvern, UK). Infrared (IR) spectra were recorded using a Perkin Elmer Spectrum 400 FTIR. Using PerkinElmer software, a UV–visible spectrophotometer (PerkinElmer, Waltham, MA, USA, Lambda 35 UV/VIS Spectrometer) was used to measure the UV absorbance of conjugates between 400 and 200 nm and 562 nm. The Australian Microscopy & Microanalysis Research Facility, Centre for Microscopy and Microanalysis, The University of Queensland (UQ), is where transmission electron microscopy (TEM; HT7700 Exalens, HITACHI Ltd., Tokyo, Japan) was performed. ELISA optical density (OD) data at 450 nm were read using a spectraMAX 250:96-well plate reader (Molecular Devices, San Jose, CA, USA). With the aid of GraphPad Prism 8.3.1, all data were examined. Using a PowerWave XS Microplate Reader from Bio-Tek (Winooski, VT, USA), the well absorbance for the cytotoxicity analysis was measured at 580 nm.

### 5.2. Synthesis of Peptide Antigen

**PJ8** (AKFVAAWTLKAAA-QAEDKVKQSREAKKQVEKALKQLEDKVQ, at 0.05 mmole scale) and azide-modified **PJ8** (N_3_CH_2_C(O)–PJ8, at 0.1 mmole scale) were manually synthesized using Fmoc-SPPS, as previously described [12].

### 5.3. Synthesis of Rods–PJ8 and Worms–PJ8

**Rod–** and **worm–PJ8** conjugates were obtained as previously reported [12].

### 5.4. Synthesis of Spheres–PJ8 and Tadpoles–PJ8

Functionalization of **spheres** and **tadpoles** with azide-modified **PJ8** was achieved by copper-catalyzed alkyne–azide “click” reaction following a procedure similar to that reported for **rods–PJ8** and **worms–PJ8** [12]. In MilliQ water, azide-modified **PJ8** (1.2 equiv, 0.68 mg per 200 µL) was dissolved and argon-purged for five minutes. **Spheres** and **tadpoles** (1 equiv, 1 mg) were dispersed in Milli-Q water (200 µL) in Eppendorf tubes, kept in an ice bath for 2 h, and then purged with argon for 5 min. Aqueous solutions of copper (II) sulfate (8.8 equiv, 0.2 mg in 100 µL) and ascorbic acid (17.6 equiv, 0.3 mg in 200 µL) were mixed together and purged with argon for 5 min. Thereafter, all solutions were combined in one flask and agitated at 200 rpm at room temperature for 8 h while protected from light. Upon reaction completion, using dialysis tubing with a molecular weight cut-off of 10 kDa, the reaction mixtures of **spheres–PJ8** and **tadpoles–PJ8** were dialyzed against MilliQ water for 72 h; the water was changed three times each day. The final product was concentrated to 1 mg/mL via nitrogen blowing. To confirm chemical conjugation, UV-vis spectroscopy was employed to measure the absorbance values of **spheres–PJ8** and **tadpoles–PJ8** solutions (Supporting Information, Figure S1). To further confirm the formation of conjugates, freeze-dried **spheres–PJ8** and **tadpoles–PJ8** were analyzed using a ninhydrin staining test (Supporting Information, Figure S2) and IR spectroscopy (Supporting Information, Figure S3). A bicinchoninic acid (BCA) protein assay was performed to quantify the amount of **PJ8** conjugated to **sphere**- and **tadpole**-shaped polymers via a previously reported and validated protocol [12] (Supporting Information, Figure S4).

### 5.5. Preparation of Physical Mixtures of Polymeric Nanoparticles and PJ8

Physical mixtures, **rods + PJ8**, **worms + PJ8**, **spheres + PJ8**, and **tadpoles + PJ8** (1:1 ratio of MW) were prepared by mixing each polymer (54 µg) with the antigen (30 µg) in PBS (oral delivery) or water (nasal delivery). All conjugates and physical mixtures were freshly prepared before testing.

### 5.6. Characterization of Vaccine Candidates

Using a Zetasizer, the average particle size and polydispersity index (PDI) of the vaccine candidates were measured by DLS in folded capillary cuvettes at a back-scattering angle of 173° at 25 °C. All the polymers, antigens, conjugated compounds, and physical mixtures were dissolved in PBS at a concentration of 30 μg/mL, which was the same as that utilized in the animal experiments. For each batch, the results were expressed as the average of at least five measurements (Supporting Information, Figure S5).

The surface morphology of the conjugated chemicals, polymers, antigen, and physical mixtures dissolved in PBS were all visualized using TEM. A drop of each sample was placed on a glow-discharged carbon-coated grid, and the particles were given two minutes to settle. The particles were dyed for two minutes with 2% uranyl acetate (pH 7). Before taking the images, the excess stain solution was wrung off and the grid was allowed to air dry for five minutes.

### 5.7. Immunological Assessment

Immunological assessment was carried out using six-week-old black/female C57 mice obtained from the Animal Resource Centre (Perth, Western Australia). Before being utilized in experiments, mice were kept in sterile cages and given seven days to become acclimated to their surroundings. The mice were divided into 20 experimental groups (10 for oral studies; 10 for intranasal studies), with five mice per group.

### 5.8. Oral Immunization Study

On day 1, all mice were given an oral dose of a freshly prepared vaccine candidate or controls. Vaccine candidates contained antigen (30 µg) in 50 µL of PBS. The mice in the positive control group were given 10 µg of cholera toxin B (CTB) along with the antigen (30 µg). For the mice in the negative control group, 50 µL of PBS was given. On days 14, 28, and 42, mice received three boosts of the same dose. On days 0, 13, 27, and 41, blood samples were collected via tail bleed; on day 56, blood samples were collected through heart puncture. The samples were centrifuged for 10 min at 4500× *g*, and the supernatant serum was removed. Saliva samples were also collected on days 0, 7, 21, 35, and 49. To induce the production of saliva, mice were administered with an intraperitoneal injection of 50 µL of a 0.1% pilocarpine solution. After that, saliva was collected and stored in tubes pretreated with the protease inhibitor phenylmethylsulfonylfluoride (PMSF). All serum and saliva samples were stored at −80 °C until further analysis.

### 5.9. Intranasal Immunization Study

On day 1, all mice received an intranasal dose of vaccine candidates (15 µL/nare) that were freshly prepared and consisted of 30 µg of antigen, either conjugated or physically mixed, with 55 µg of polymeric nanoparticles in 30 µL of endotoxin-free water. Mice in the positive control group received CTB (10 µg) and antigen (30 µg). Mice in the negative control group were administered with 50 µL of endotoxin-free water. Two boosts of the same doses were given to each mouse at intervals of two weeks. On days 0, 13, and 27, blood samples were collected via tail bleed; on day 41, blood samples were collected through heart puncture. The samples were centrifuged for 10 min at 4500× *g*, and the supernatant serum was removed. On days 0, 7, 20, and 34, saliva samples were collected using the same procedure as above. All serum and saliva samples were stored at −80 °C until further analysis.

### 5.10. Determination of Antibody Titers

Saliva and post-immunization sera were tested for the presence of J8-specific antibody IgG titers using ELISA. To reduce nonspecific binding, 96-well microtiter plates were first coated with carbonate coating buffer (CCB) containing 50 µg of J8 per plate and then blocked with 5% skim milk. Starting with a serum concentration of 1:100 and a saliva concentration of 1:2, two-fold serial dilutions were performed using 0.5% skim milk on the antigen-coated plates. naïve mouse sera and saliva (day 0 samples) were employed as controls. Then, the secondary antibody was applied at dilution: 1:3000 IgG-HRP for serum and 1:1000 IgG- or IgA-HRP for saliva. Following that, plates were incubated for 20 min at room temperature with 100 µL (per well) of O-phenylenediamine dihydrochloride. The absorbance was measured with a Spectra Max Microplate reader at 450 nm. Antibody titers were defined as the lowest dilution with an optical density greater than the mean absorbance plus three standard deviations of control wells (pre-immunization, day 0 sera/saliva). One-way ANOVA with Tukey’s multiple comparison test was used for statistical analysis.

### 5.11. Indirect Bactericidal Assay

The bactericidal tests were completed as previously reported [12].

### 5.12. In Vitro Stability against Proteolysis

An enzyme 1000 U solution was prepared using bovine trypsin (10,000 U/mg) and 1 mM CaCl2 in 1 mL PBS [39,40]. Then, antigen solution (400 µL), free and/or conjugated with **PJ8** peptide (1.25 mg/mL) in PBS, was mixed with enzyme solution (100 µL). The mixture was shaken and incubated at 37 °C. At desired time points, aliquots of the peptide solution (60 µL) were mixed with 80 µL of the TFA and acetonitrile mixture (1:2) to inhibit the activity of the enzyme. The remaining (undigested) starting material was analyzed by HPLC using the calibration curve.

### 5.13. Ethics Statement

This study was performed according to strict regulations from the National Health and Medical Research Council (NHMRC) of Australia (Australian Code of Practice for the Care and Use of Animals for Scientific Purposes, 8th edition 2013). All animal procedures and protocols were approved by The University of Queensland Animal Ethics Committees (AECs), AEC Approval Number: SCMB/AIBN/069/17.

## Data Availability

Additional data available in Supplementary Material and upon request from the authors.

## Supplementary Materials

The following supporting information can be downloaded at: https://www.mdpi.com/article/10.3390/vaccines12020198/s1, Figure S1: UV absorbance of nanoparticles; Figure S2: Ninhydrin staining of nanoparticles; Figure S3: IR spectrum of nanoparticles; Figure S4: Quantification of PJ8 conjugation; Figure S5: DLS spectra of particles; Figure S6: The nanoparticles’ morphology examined by transmission electron microscopy; Figure S7: The relationship between IgG titers and opsonization/bactericidal against GC2203 GAS strain; Table S1: Size and PDI of nanoparticles analyzed by DLS.

## Abbreviations

GAS, Group A *Streptococcus*; iGAS, invasive GAS; RHD, rheumatic heart disease; PADRE, pan HLA DR-binding epitope; CTB, cholera toxin B; ELISA, enzyme-linked immunosorbent assays; PDI, polydispersity index; DLS, dynamic light scattering; TEM, transmission electron microscopy; CD, circular dichroism; CFA, complete Freund’s adjuvant; IgG, immunoglobulin G; IgA, immunoglobulin A; CFU, colony-forming units; OD, optical density; TFA, trifluoroacetic acid; Fmoc, fluorenylmethyloxycarbonyl; PBS, phosphate-buffered saline, BCA, bicinchoninic acid.
