# Supplementary material for: Polymeric Nanoparticles as Oral and Intranasal Peptide Vaccine Delivery Systems: The Role of Shape and Conjugation
Source: Vaccines (Basel). 2024 Feb 15;12(2):198. doi: 10.3390/vaccines12020198 (PMC10893271; doi:10.3390/vaccines12020198)
Supplement: Supplementary file 1 [file vaccines-12-00198-s001.zip › vaccines-2860093-supplementary.pdf]

Polymeric Nanoparticles as Oral and Intranasal Peptide Vaccine Delivery Systems: The Role of Shape and Conjugation

Prashamsa Koirala [1], Ahmed O. Shalash [1], Sung-Po R. Chen [2], Mohammad O. Faruck [1], Jingwen Wang [1], Waleed M. Hussein [1], Zeinab G. Khalil [3], Robert J. Capon [3], Michael J. Monteiro [2], Istvan Toth [1,4,*] and Mariusz Skwarczynski [1,*]

[1] School of Chemistry and Molecular Biosciences, The University of Queensland, Brisbane, QLD 4072, Australia

[2] Australian Institute of Bioengineering and Nanotechnology, The University of Queensland, Brisbane, QLD 4072, Australia

[3] Institute for Molecular Bioscience, The University of Queensland, St Lucia, QLD 4072, Australia

[4] School of Pharmacy, The University of Queensland, Woolloongabba, QLD 4102, Australia

* Correspondence: i.toth@uq.edu.au (I.T.); m.skwarczynski@uq.edu.au (M.S.)

Table S1. Size and PDI of nanoparticles analysed by DLS. Characterizations of **rods** and **worms** alone, in the mixture and as the conjugates are adopted from the published report.[1]

Nanoparticles	Detected size	PDI
**Sphere**	90 ± 5	0.16 ± 0.01
**Sphere+PJ8**	460 ± 11	0.12 ± 0.04
**Sphere-PJ8**	490 ± 8	0.14 ± 0.01
**Rods**	70 ± 25; 330 ± 20; 4,340 ± 10	0.30 ± 0.01
**Rods+PJ8**	78 ± 12; 440 ± 30; 2,000 ± 1,000	0.30 ± 0.05
**Rods-PJ8**	66 ± 6; 450 ± 20; 4,800 ± 400	0.40 ± 0.01
**Worms**	350 ± 9; 2,650 ± 10	0.40 ± 0.04
**Worms+PJ8**	600 ± 22; 5,000 ± 400	0.30 ± 0.03
**Worms-PJ8**	550 ± 10; 4,000 ± 2000	0.40 ± 0.03
**Tadpoles**	200 ± 40; 1,900 ± 100; 4,380 ± 40	0.27 ± 0.01
**Tadpoles+PJ8**	200 ± 4	0.20 ± 0.01
**Tadpoles-PJ8**	190 ± 7	0.21 ± 0.01

a)

b)

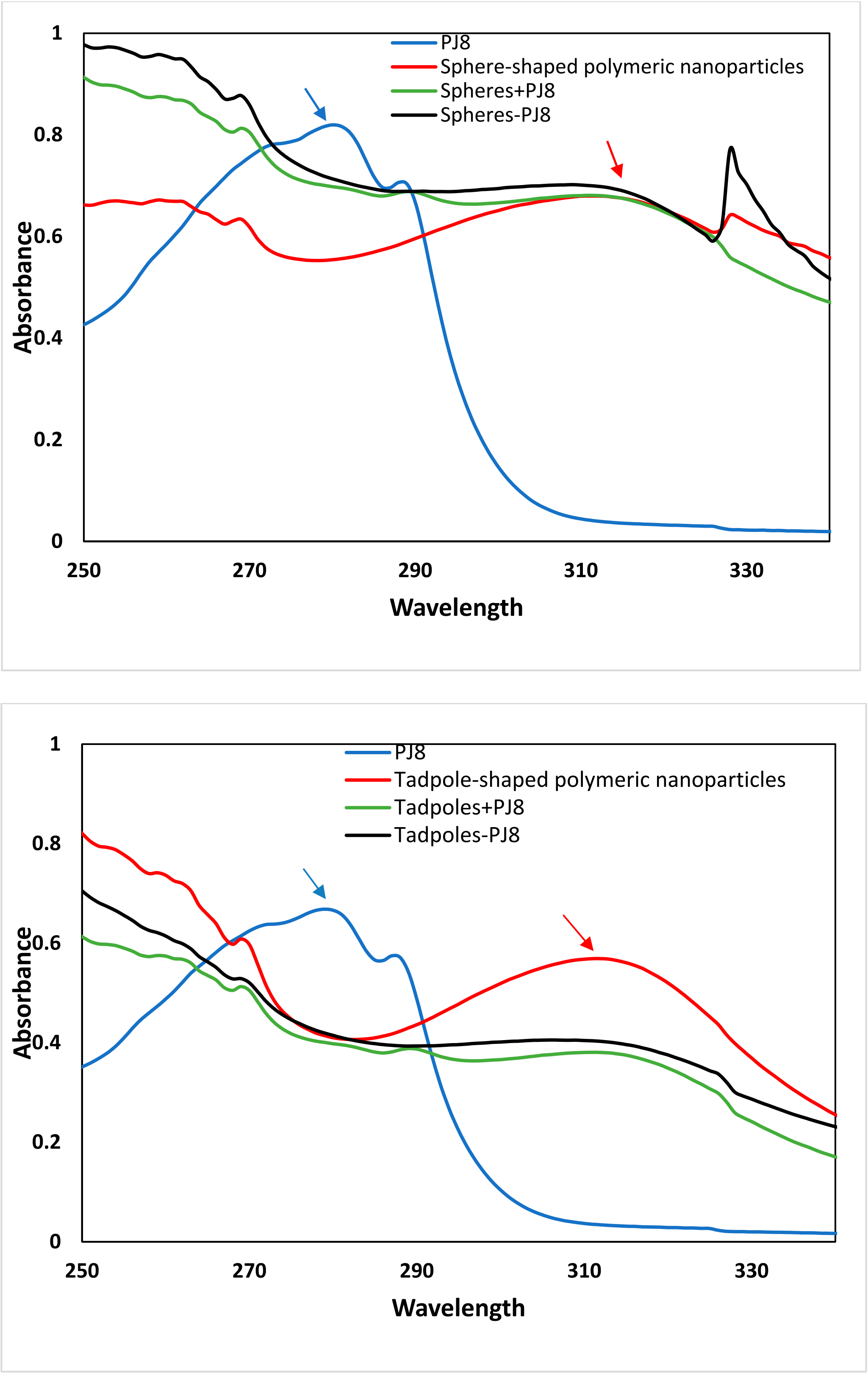

Figure S1. UV absorbance of a) **PJ8**, **spheres** polymeric nanoparticles, **spheres-PJ8**, **spheres+PJ8**; and b) **PJ8**, **tadpoles** polymeric nanoparticles, **tadpoles-PJ8**, **tadpoles+PJ8** from 200 nm to 350 nm. Blue arrows denote the absorbance peak observed at 280 nm and red arrows denote the absorbance peak observed at 312 nm.

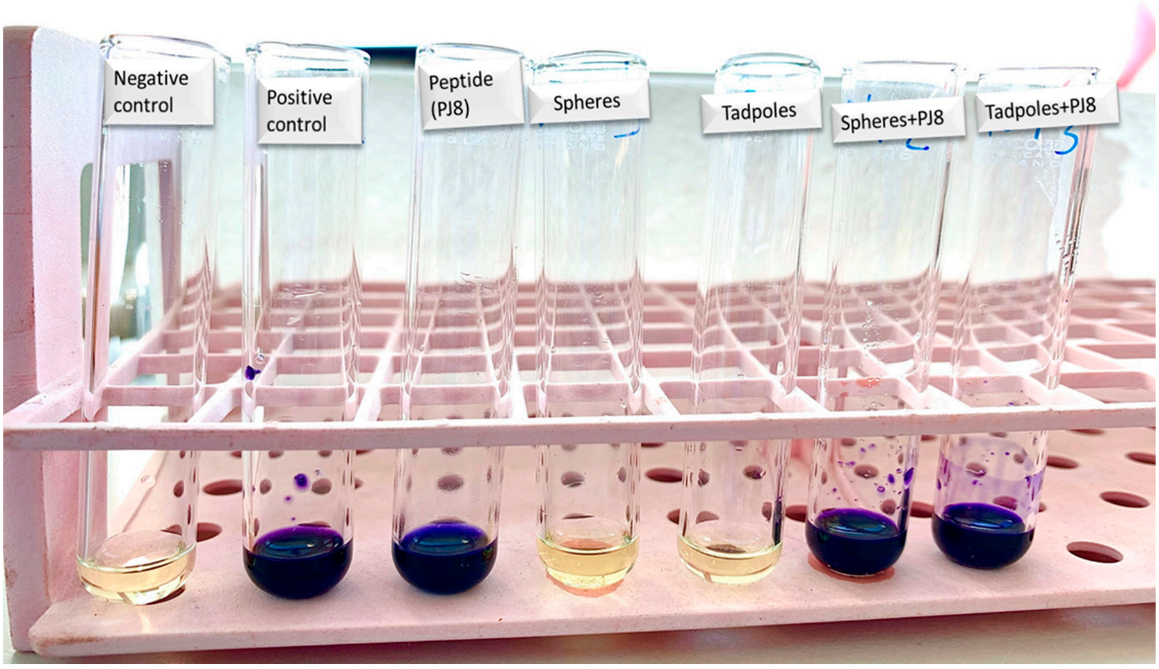

Figure S2. Ninhydrin staining test to confirm the formation of **spheres-PJ8** and **tadpoles-PJ8.**

a)

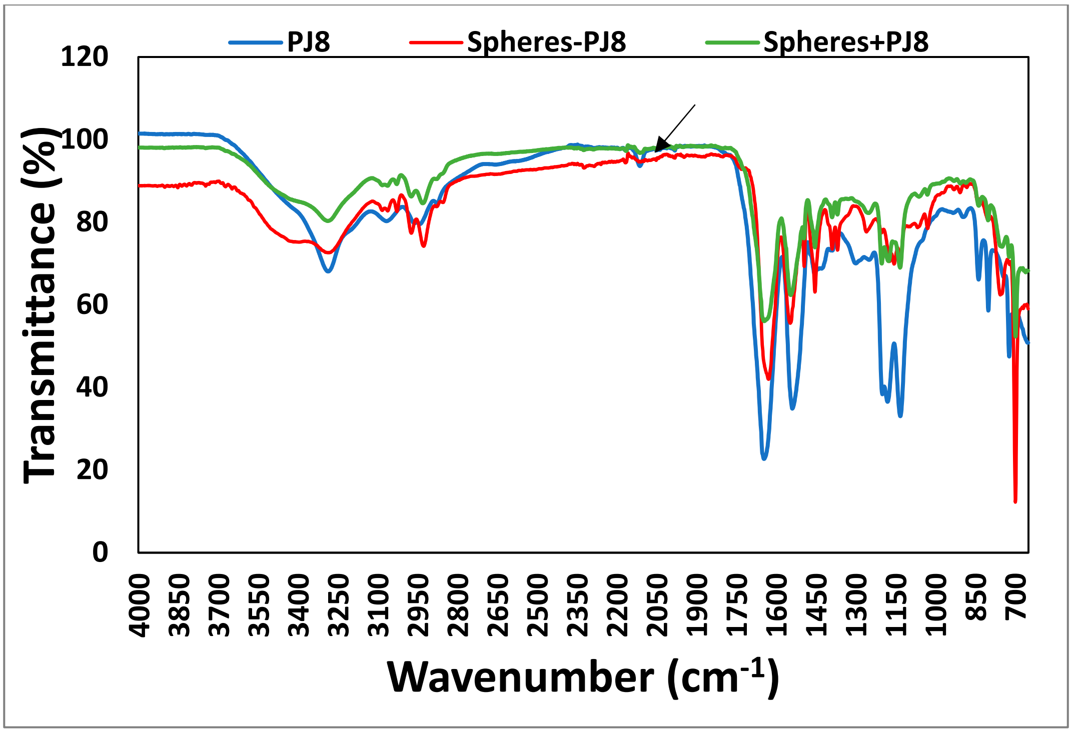

b)

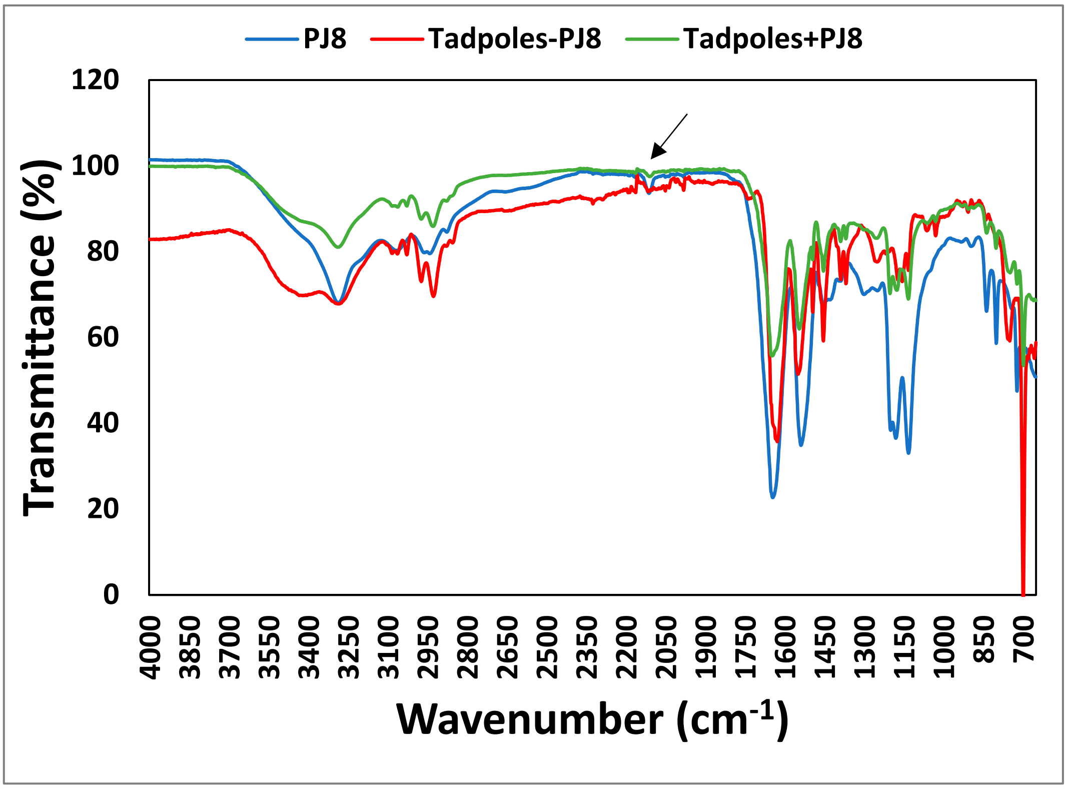

Figure S3. IR spectrum of a) Azide functionalised **PJ8**, **sphere** polymeric nanoparticles, and **spheres+PJ8** b) Azide functionalised **PJ8**, **tadpole** polymeric nanoparticles, and **tadpole+PJ8.** Arrows denote a characteristic azido stretching vibration observed at 2111 cm^{-1} for azide functionalised **PJ8** and the physical mixture of **PJ8** and **spheres** and **tadpoles**.

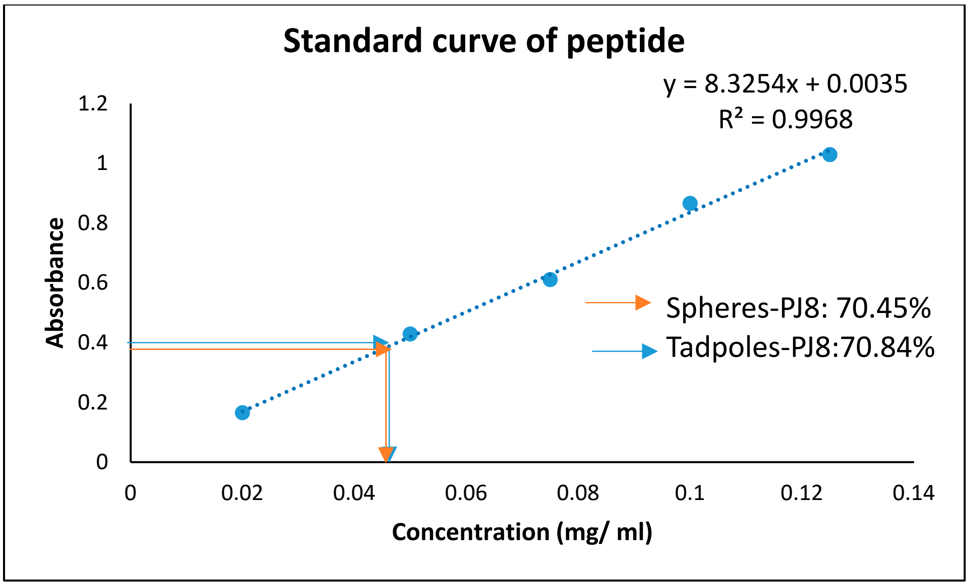

Figure S4. Quantification of **PJ8** conjugated to **sphere** and **tadpole** polymeric nanoparticles via BCA asssay.

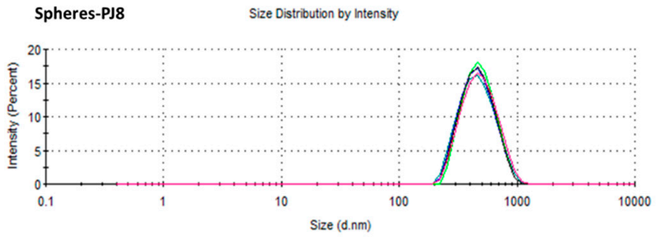

Particle size	PDI
Peak 1: 490 ± 8	0.14 ± 0.01

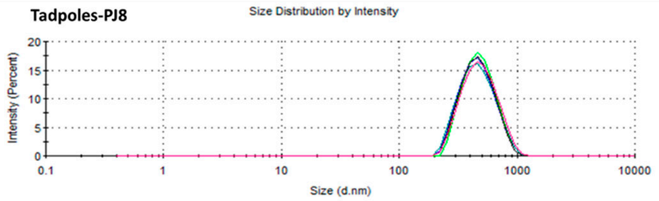

Particle size	PDI
Peak 1: 190 ± 7	0.21 ± 0.009

Figure S5. DLS spectra of particles **spheres-PJ8** and **tadpoles-PJ8,** size distributions by intensity.

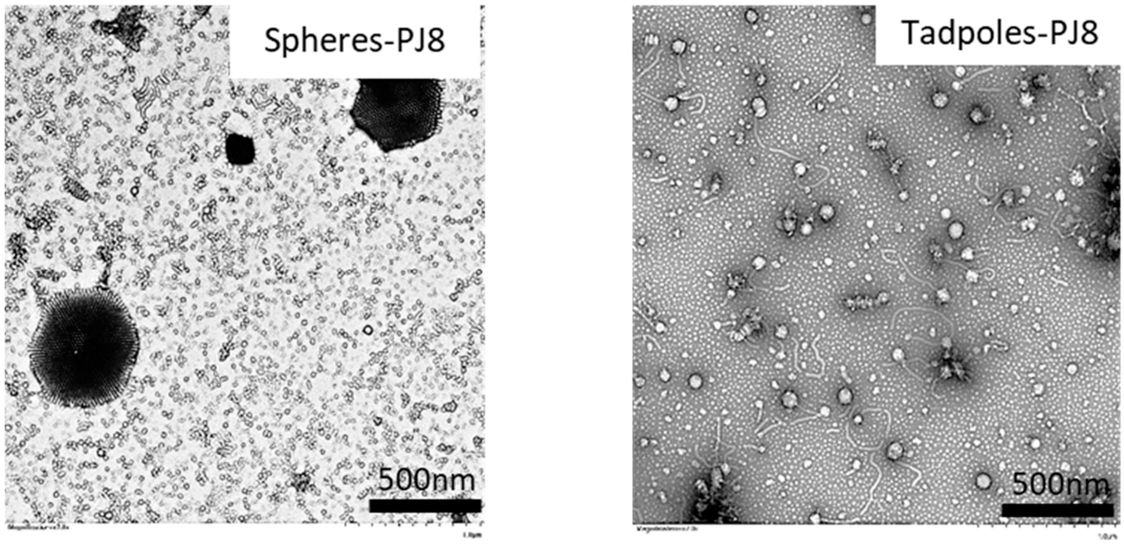

Figure S6. The nanoparticles' morphology examined by transmission electron microscopy. TEM images of **spheres-PJ8** and **tadpoles-PJ8** at 0.1 mg/mL. The samples were treated with 2% uranyl acetate as a negative stain (scale bars: 500 nm).

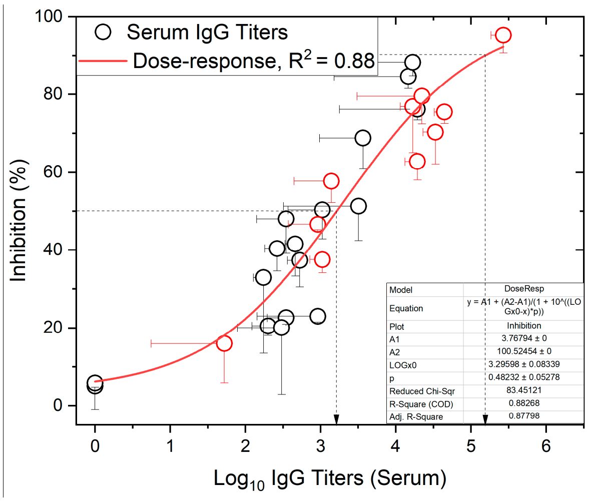

Figure S7. The relationship between anti-J8 IgG titers in sera of mice and opsonization/bactericidal activity against cultured GAS bacteria (GC2203 strain). The data were fitted to a sigmoidal relationship, gave R^2 of 0.88. Red circles denote subcutaneous immunization,[1] while black circles denote intranasal and oral immunization. Dashed arrows represent the interpolated IC_{50} and IC_{90} titer values from the fitted curve.

1. Koirala, P.; Chen, S.-P.R.; Boer, J.C.; Khalil, Z.G.; Deceneux, C.; Goodchild, G.; Lu, L.; Faruck, M.O.; Shalash, A.O.; Bashiri, S., et al. Polymeric Nanoparticles as a Self-Adjuvanting Peptide Vaccine Delivery System: The Role of Shape. *Adv. Funct. Mater.* **2023**, https://doi.org/10.1002/adfm.202209304, 2209304, doi:https://doi.org/10.1002/adfm.202209304.
